# Assessing and genotyping threatened staghorn coral *Acropora cervicornis* nurseries during restoration in southeast Dominican Republic

**DOI:** 10.7717/peerj.8863

**Published:** 2020-04-17

**Authors:** Johanna Calle-Triviño, Renata Rivera-Madrid, María Geovana León-Pech, Camilo Cortés-Useche, Rita Inés Sellares-Blasco, Margarita Aguilar-Espinosa, Jesús Ernesto Arias-González

**Affiliations:** 1 Departamento de Recursos del Mar, Centro de Investigación y de Estudios Avanzados del I.P.N., Unidad Mérida, Yucatán, México; 2 Wave of Change Iberostar Hotels & Resorts, Quintana Roo, Mexico; 3 Unidad de Bioquímica y Biología Molecular de Plantas, Centro de Investigación Científica de Yucatán A.C., Mérida, Yucatán, México; 4 Department of Biological Science, University of Rhode Island, Kingston, RI, USA; 5 Fundación Dominicana de Estudios Marinos, Bayahíbe, Dominican Republic

**Keywords:** *Acropora cervicornis*, Coral reef restoration, Coral nurseries, Outplanted colonies, Threatened staghorn coral, Survival, Productivity, Growth, Genetic characterization, Dominican Republic

## Abstract

*Acropora cervicornis* is a structurally and functionally important Caribbean coral species. Since the 1980s, it has suffered drastic population losses with no signs of recovery and has been classified as a critically endangered species. Its rapid growth rate makes it an excellent candidate for coral restoration programs. In 2011, the Fundación Dominicana de Estudios Marinos (Dominican Marine Studies Foundation, FUNDEMAR) began an *A*. *cervicornis* restoration program in Bayahibe, southeast Dominican Republic. In this study, we present the methodology and results of this program from its conception through 2017, a preliminary analysis of the strong 2016 and 2017 cyclonic seasons in the greater Caribbean, and a genetic characterization of the “main nursery”. The mean survival of the fragments over 12 months was 87.45 ± 4.85% and the mean productivity was 4.01 ± 1.88 cm year^−1^ for the eight nurseries. The mean survival of six outplanted sites over 12 months was 71.55 ± 10.4%, and the mean productivity was 3.03 ± 1.30 cm year^−1^. The most common cause of mortality during the first 12 months, in both nurseries and outplanted sites, was predation by the fireworm, *Hermodice carunculata*. We identified 32 multilocus genotypes from 145 total analyzed individuals. The results and techniques described here will aid in the development of current and future nursery and outplanted site restoration programs.

## Introduction

Comprised of 368 species, the genus *Acropora* is the world’s most abundant coral group. Of these species, only *Acropora cervicornis, Acropora palmata*, and the hybrid *Acropora prolifera* are found in the Caribbean and Western Atlantic (National Marine Fisheries Service, 2015). Historically, *A. cervicornis* and *A. palmata* have dominated the region, building shallow reefs (Garzón-Ferreira, Moreno-Bonilla & Valderrama, 2004) with branched structures that provide crucial habitats for reef organisms. The interactions and complex flows of energy around these species lead to high levels of primary productivity and interspecies interactions (Itzkowitz, 1978; Lirman, 1999; Bruckner, 2002).

The early 1980s saw a loss of up to 97% of both *A. cervicornis* and *A. palmata* cover caused by several factors: white band disease, hurricanes and storms, corallivorous predation, thermal stress, pollution, and, in the case of *A. palmata*, mean sea level increase (Gladfelter, 1982; Porter, Battey & Smith, 1982; Knowlton, 1992; Hughes, 1994; Aronson et al., 2002; Miller, Bourque & Bohnsack, 2002; Bruckner, 2002). To this day, these issues continue to prevail with no significant signs of recovery (Aronson & Precht, 2001; Chamberland, Petersen & Vermeij, 2013). Both species have been listed as critically endangered by the International Union for Conservation of Nature (IUCN) (Aronson et al., 2008) and included in Appendix II of the Convention on International Trade in Endangered Species of Wild Fauna and Flora (CITES). Proposed recovery efforts for this genus at regional and local levels have included the implementation of Marine Protected Areas (MPAs), coral restoration, and the control of coral-degrading terrestrial sources of pollution (Rinkevich, 2000; Bruckner & Hourigan, 2002; Miller & Szmant, 2006; Herlan & Lirman, 2008; Lirman et al., 2010; Toh, Ng & Chou, 2013). Pioneering work on coral restoration began in the 1970s and 1980s in the Indo-Pacific Ocean and Red Sea (Maragos, 1974; Bouchon, Jaubert & Bouchon-Navaro, 1981; Abelson, 1982; Harriott & Fisk, 1988). In the 1990s, these same areas saw the first large-scale restoration projects (Rinkevich, 1995; Oren & Benayahu, 1997; Treeck & Schuhmacher, 1997), and a coral gardening technique was soon implemented. In the initial phase, coral are grown at an in situ nursery and are outplanted in the second phase. Coral gardening has higher success rates than direct transplanting because it avoids mechanical damage, predation, and competition for space with nurseries during propagation (Bowden-Kerby, 2001; Epstein, Bak & Rinkevich, 2003; Shafir, Van Rijn & Rinkevich, 2006; Shafir & Rinkevich, 2008; Shaish, Abelson & Rinkevich, 2007; Arias-González et al., 2015). *Acropora* coral gardening restoration started in the 1990s and 2000s in Puerto Rico (Bowden-Kerby, 2001; Hernández-Delgado, Rosado-Matías & Sabbat, 2001). In their literature review, Young, Schopmeyer & Lirman (2012) reported on 60 projects working with *Acropora* across 14 Caribbean countries: 48% of studies worked with *A. cervicornis*, 12% with *A. palmata*, and 40% with both species. Lirman & Schopmeyer (2016) reported on more than 150 programs in over 20 Caribbean countries. However, few published studies have focused on the long-term success of restoration projects since it is difficult to assess the performance of propagation efforts (Griffin et al., 2012; Lirman et al., 2014; Schopmeyer et al., 2017).

In the Dominican Republic, *A. cervicornis* is disappearing from areas where it was once common (Chiappone, 2001; Geraldes & Vega, 2002; Weil, 2006; Lirman, 2010; Cortés-Useche et al., 2019). In 2011, the Fundación Dominicana de Estudios Marinos (Dominican Marine Studies Foundation, FUNDEMAR) created the Coral Restoration Program in Bayahibe which is located on the southeastern part of the island. Initially, the restoration program worked with *A. cervicornis* fragments from one of the Punta Cana Ecological Foundation (FEPC) nurseries and fragments rescued from the Bayahibe area. Later, the first nursery was expanded and new nurseries were created to take advantage of the species’ fast growth and high survival rate.

Our study offers a temporal assessment of the coral restoration program since its implementation in 2011–2017, in addition to a preliminary analysis of the strong cyclonic seasons that struck the Greater Caribbean region in 2016 and 2017.

The two main objectives of this study were: (1) to assess the coral restoration program over time, analyzing the results within the context of the “regional restoration benchmarks for *A. cervicornis”* proposed by Schopmeyer et al. (2017); and (2) to determine the genetic diversity of *A. cervicornis* colonies in the “main nursery” for use in future regional restoration efforts.

## Materials and Methods

### Study location

**Figure 1:**
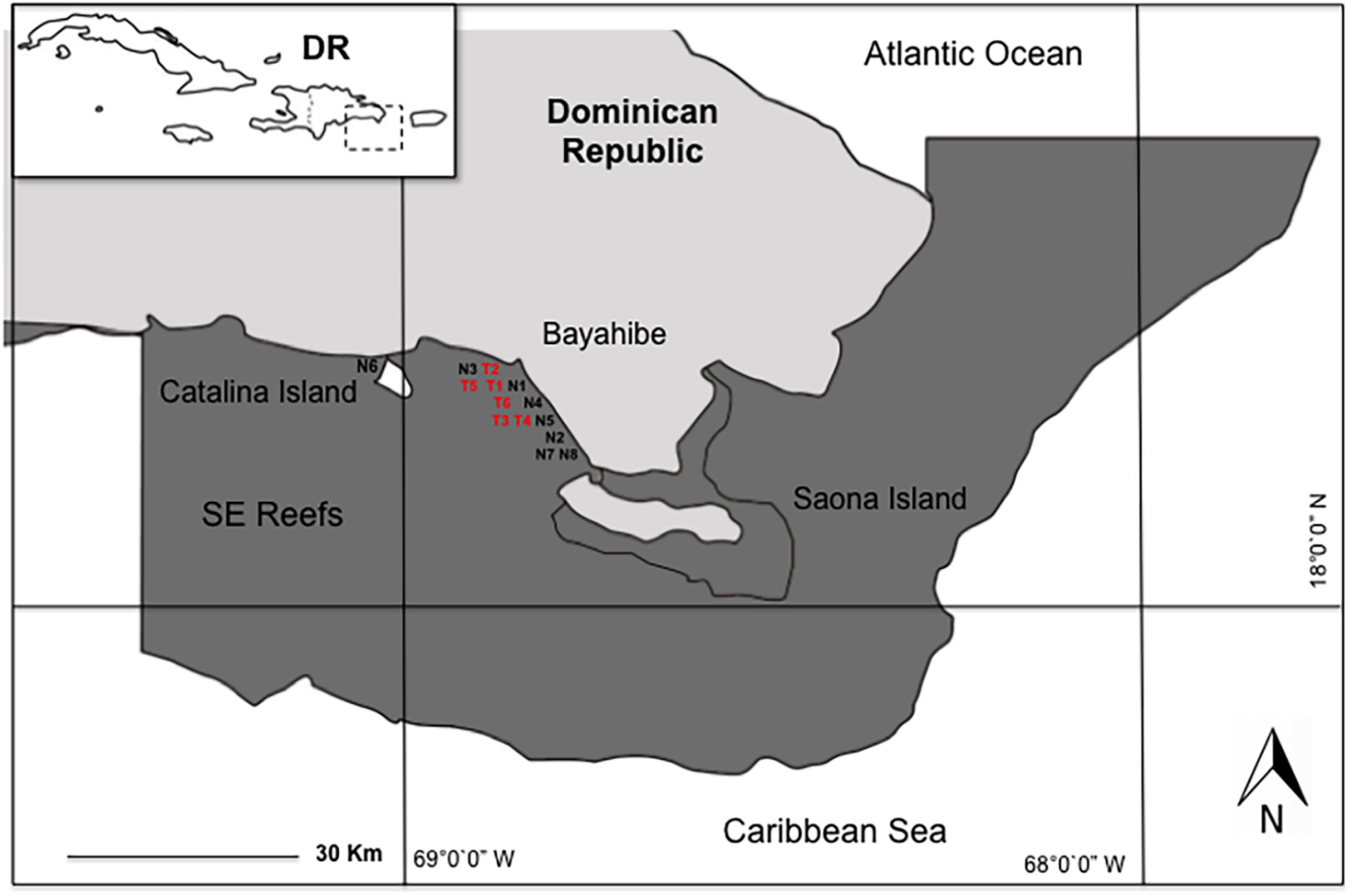
Study area in Bayahibe coast, Dominican Republic. Nurseries: FUNDEMAR-N1 = “mother nursery”, Catalonia-N2, Dreams-N3, Scuba fun-N4, Viva-N5, Catalina-N6, Iberostar-N7, Canoa-N8. Outplanting reefs: FUNDEMAR-T1, Coralina-T2, Pepito I-T3, Pepito II-T4, Atlantic Princess-T5, Costa Romántica-T6.

We conducted the study in eight coral nurseries and six outplanting areas in the Southeast Reefs Marine Sanctuary (Santuario Marino Arrecifes del Sureste) located in the Dominican Republic’s Bayahibe municipality along the southeastern Caribbean coast. This area was declared a Marine Protected Area (MPA) by decree 571-09 on August 7, 2009 (Fig. 1). This MPA attracts 2,000–2,500 tourists daily, with an annual average of 600,000 visitors and generating a 250 million dollar revenue. Tourism’s impact on coastal marine ecosystems is significant (Ministry of Environment & Natural Resources, 2012; Cortés-Useche et al., 2018), mainly due to local stress factors such as the constant flow of boats and visitors, snorkeling activities, water sports, and “artisanal” fishing (Cortés-Useche et al., 2019).

### Nurseries and outplanted establishments

The pilot project began in 2011 with one nursery, the “main nursery”, comprised of four structures, each supporting approximately 30 fragments. Most of the *A. cervicornis* fragments came from one of the FEPC’s nurseries (with multiple genotypes collected in Punta Rusia, Samaná, Bávaro, Punta Cana, and La Caleta National Submarine Park), while other fragments were collected from the Bayahibe region. Since its beginning, the restoration program’s design involved the local community, and included local volunteers such as fishermen, boat captains, tourism service providers, park rangers, diving instructors, divers, university students, and hotel owners. All received comprehensive training and contributed their time, equipment, materials, and boats at different developmental stages of the restoration program.

**Figure 2:**
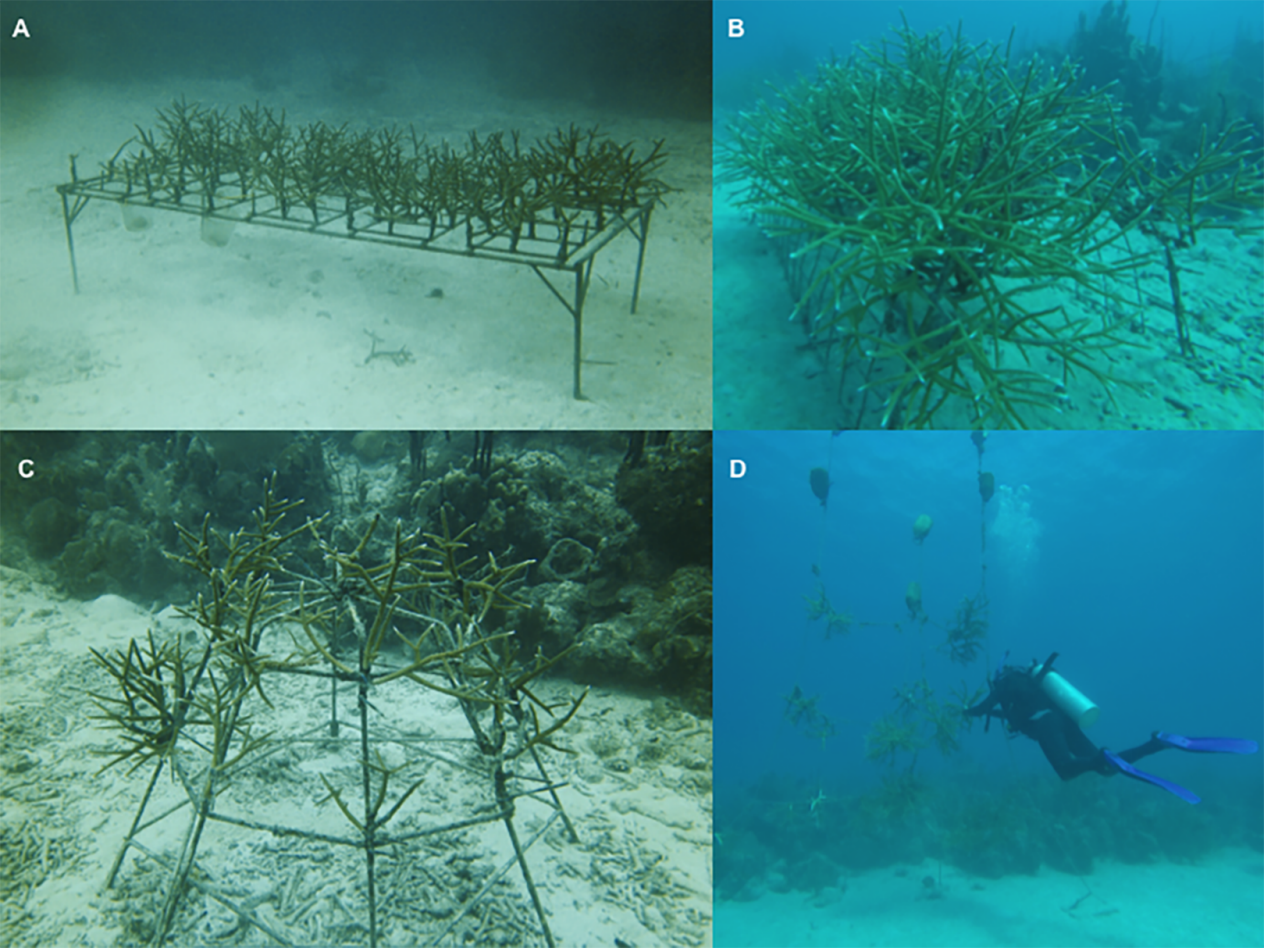
Structures used in the Bayahibe nurseries. (A) Table type structure, capacity for 50 fragments (B) Frame type structure, capacity for 30 fragments (C) Dome type structure, capacity for 20 fragments (D) Rope type structure, capacity for 30 fragments.

In 2012, the main nursery was expanded using 2nd and 3rd generation corals propagated within the nursery (FUNDEMAR-N1). Twenty-two structures, holding > 600 fragments, were added. Seven frames were built with welded electromesh measuring 1.30 m long and 2 m wide, eight domes, a table build with 1/2" Ø metal corrugated rod approximately 1 m high, and six ropes 5.5 m high and 2 m wide (Fig. 2). This nursery was the prototype for the remaining seven nurseries and six outplanted areas that were in use until 2017.

**Figure 3:**
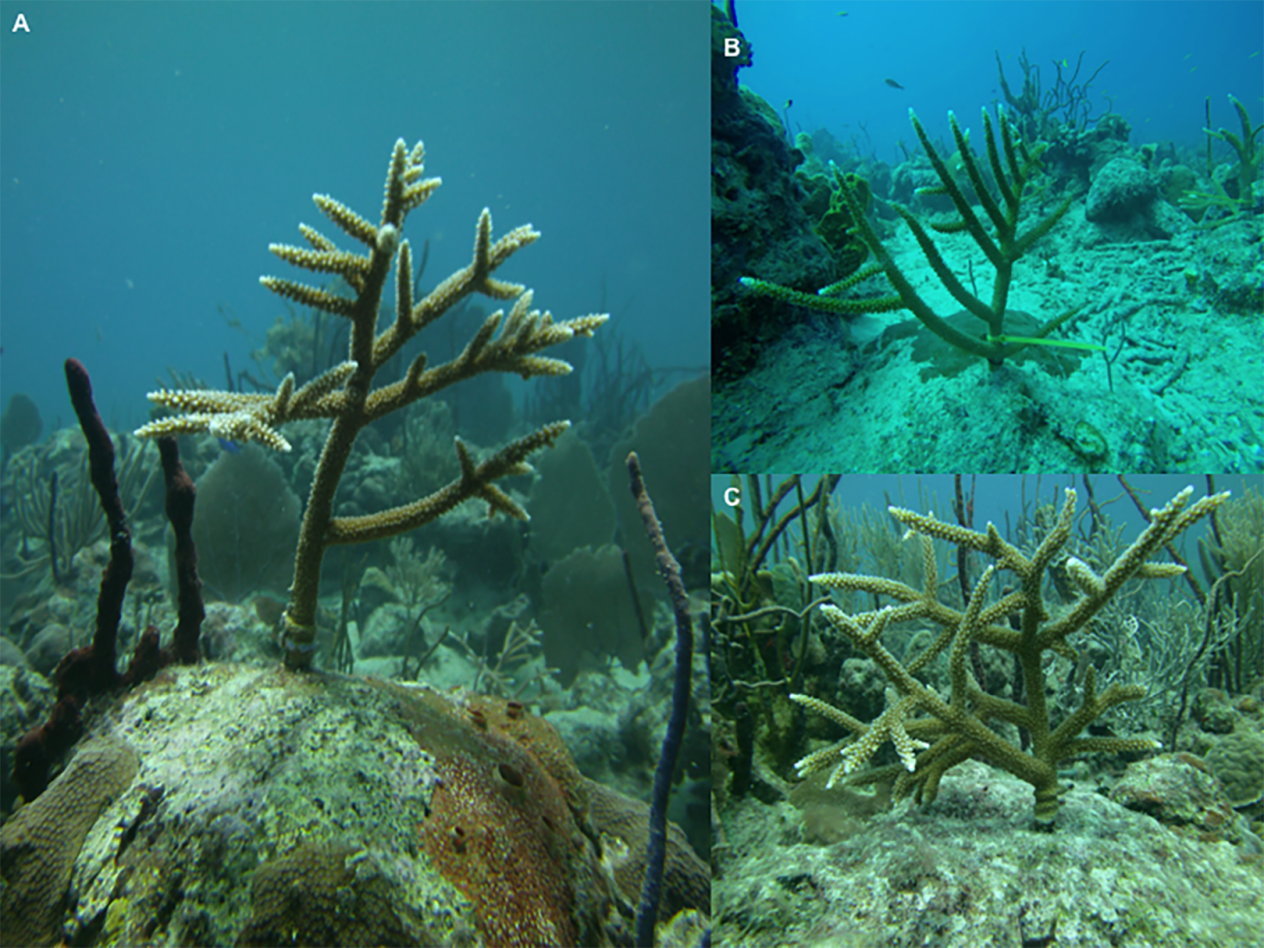
(A) *Acropora cervicornis* outplanting sites (B) Coral colonies attached to nails with plastic straps (C) Tissue covering straps on the base of the coral.

Subsequent dives were initiated to select outplanted sites, which were chosen based on the following criteria: depth, presence of wild *Acropora* colonies, low sedimentation, low macroalga cover, and the presence of calcareous coral algae (CCA) (Edwards, 2010; Johnson et al., 2011; Mercado Molina et al., 2013; Arias-González et al., 2015; Carne, Kaufman & Scavo, 2016). Before outplanting, the substrate was cleaned using different hand tools (brushes, chisels, hammers) to remove algal mats, sediments, or macroalgae, but the CCA was left alone. Once the substrate was prepared, steel nails were driven directly into the substrate, leaving an approximate distance of 0.5–1 m between the nails (Mercado Molina et al., 2013). Plastic straps were used to attach the coral colonies as tightly as possible to the nails (Johnson et al., 2011) to prevent ocean currents from causing friction or loosening the nails (Fig. 3). Over time, coral tissue covered the straps and the colonies healed completely.

In 2013, the first two outplanted projects were carried out across zones T1 and T3 for a total of 214 outplanted colonies. In 2014, a new nursery (N2) was installed and two more outplanted sites were established (T2 and T4) for a total of 529 *A. cervicornis* outplanted colonies. In 2015, another four nurseries were established (N3, N4, N5 and N6) and a total of 743 corals were outplanted. After Hurricane Matthew in September 2016, two sites (T3 and T4) were closed and two new outplanted sites (T5 and T6) were established in protected areas (Table 1). In 2017, a total of eight nurseries were established, with more than 26,000 cm of tissue, six outplanted sites, and 1,446 outplanted colonies (Table 2).

**Table 1:** Restoration Program: in situ *Acropora cervicornis* propagation nurseries and outplanting sites data up to 2016.

Nursery	Year established	Outplanted sites	Year established	Year outplanted sites closed
N1.FUNDEMAR	2011	T1-FUNDEMAR	2013	–
N2-CATALONIA	2014	T3- PEPITO I	2013	2016
N3-DREAMS	2015	T2-CORALINA	2014	–
N4-SCUBA FUN	2015	T4- PEPITO II	2014	2016
N5-VIVA	2015	T5-ATLANTIC PRINCESS	2016	–
N6-CATALINA	2015	T6-COSTA ROMÁNTICA	2016	–
N7-IBEROSTAR	2016	–	–	–
N8-CANOA	2016	–	–	–

**Table 2:** Number of *Acropora cervicornis* colonies outplanted per year.

Year	# Outplanted corals
2013	214
2014	529
2015	–
2016	703
Total	1,446

Each propagating coral nursery had different ropes, frames, domes, tables, and figure structures that were maintained every 2 weeks to remove coral competitors such as macroalgae, hydroids, and bivalves and predators like fireworms (Arias-González et al., 2015). Nurseries and outplanted sites had a depth of 12.5 m and occupied an area of approximately 200 m^2^ except for the N6-Catalina nursery and T2-Coralina, both of which were between 2 and 5 m deep, respectively.

### Fragments and survival metrics

We used the methodology proposed by Schopmeyer et al. (2017) to determine restoration success by evaluating the growth, survival, and productivity of colonies installed in the nurseries and outplanted sites. We monitored sites during the 12-month period after their creation to compare them with the benchmarks provided by Schopmeyer et al. (2017) for six programs in Florida and Puerto Rico. They proposed the following reference points for measuring the first year of *A. cervicornis* restoration: (1) the survival of corals in the nursery must be greater than 80%, and (2) the survival of outplanted corals must be greater than 70%. Average productivity should be >4.4 cm year^−1^ for corals in nurseries and >4.8 cm year^−1^ for outplanted corals.

Schopmeyer et al. (2017) also considered a stop-light model based on the relative performance (mean) of each nursery and outplanted zone for each restoration criteria. In this model, values within 10% of the overall mean are considered green (desirable benchmark: no actions or improvement need to be made); values between 10% and 20% below the mean are considered yellow (caution: some adjustments must be made); and values 20% below the mean are considered red (action must be taken to improve methods, design, or site selection). These measures are proposed for sites in years without large-scale disturbances such as temperature anomalies or hurricanes. The authors suggested that these reference points, and possible subsequent adaptative management, are necessary to fully evaluate the long-term success of coral restoration and species recovery programs.

Growth and survival data were taken quarterly, and coral from both nurseries and outplanted sites were individually labeled. Each of the branch fragments were measured to the nearest centimeter with a flexible ruler. Growth was expressed as Total Linear Extension (TLE) in cm (Lirman et al., 2014). The change in TLE in one year (growth) was estimated as: Total annual growth = (Final Measure-Initial Measure).

Colony survival was determined by counting the number of colonies with some percentage of living tissue at the start of the study, and then 12 months later. If a colony was completely dead (100% dead tissue), we noted the presumed cause of mortality (Lirman et al., 2014; Schopmeyer et al., 2017).

Annual productivity was determined by the following formula: Annual productivity = (growth/initial TLE). This was calculated by only grouping together fragments that were alive during the entire 12-month period that grew positively; fragments with partial tissue loss were not measured (Lirman et al., 2014; Schopmeyer et al., 2017).

### Genetic characterization

To describe the clonal diversity of the main nursery, samples were collected from three different structures: rope (60), frame (70), and dome (15). Since the corals were was not arranged or divided by potential genotype, random corals were sampled for genetic analysis. We collected 1 cm^2^ tissue samples from 145 colonies for genotyping. Collections were permitted by the Ministry of Environment and Natural Resources (permit DO-00440). Samples were placed in vials with 95% ethanol, stored, and taken to the Center for Scientific Research of Yucatan (CICY) for analysis. Once there, DNA samples were extracted using a DNeasy® Blood & Tissue Kit (Qiagen, Hilden, Germany).

We used four loci originally developed for *A. palmata* (Baums, Miller & Hellberg, 2005, Baums et al., 2009), and conditions for PCR were established according to the protocol proposed by Baums, Miller & Hellberg (2005). Forward primers were tagged with IRDye 800 or IRDye 700 (LiCor, Lincoln, NE, USA). Genotyping was performed in a DNA IR24300 sequencer (Li Cor, Lincoln, NE, USA). Finally, the diversity genetic index was estimated using GenClone software v. 2 and GenAlEx v 6.4 to discriminate distinct multilocus genotypes (MLGs).

## Results

### Survival and growth dynamics of nursery corals

**Figure 4:**
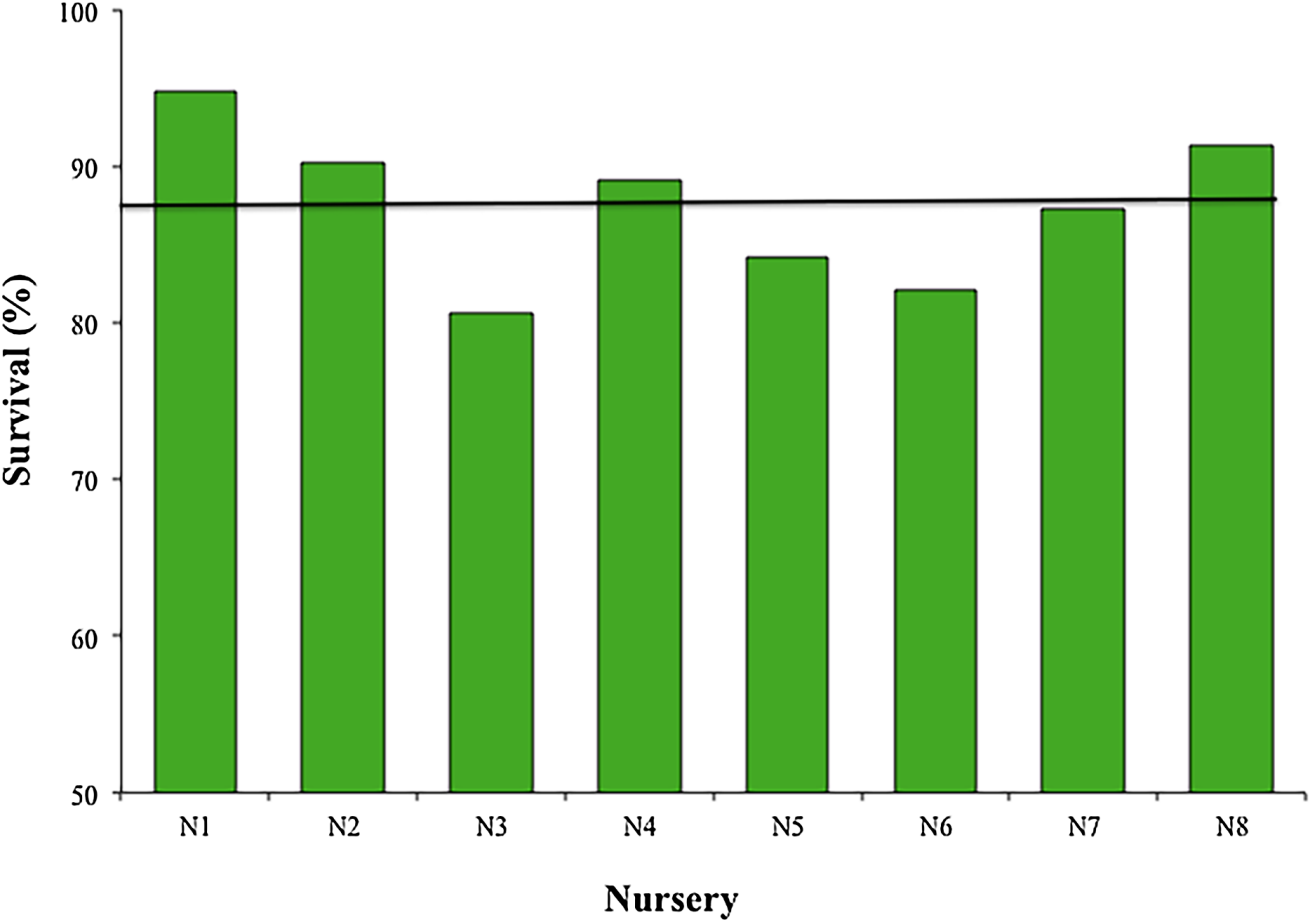
Survival percentage of *Acropora cervicornis* fragments in eight coral nurseries. Green bars indicate that they are within 10% of the overall mean, indicated by the black line.

The mean survival of the fragments during the 12 months across all nurseries was 87.45 ± 4.85%, with a range of 80.6–94.8%; sample sizes were N1, N2, N4, N5, N7, N8 = 119; N3 = 98; and N6 = 102 (Fig. 4). The most common cause of mortality in nurseries was the presence of an accelerated tissue loss syndrome. Competition from algae, sponges, and hydroids was less prevalent due to nursery maintenance practices.

**Figure 5:**
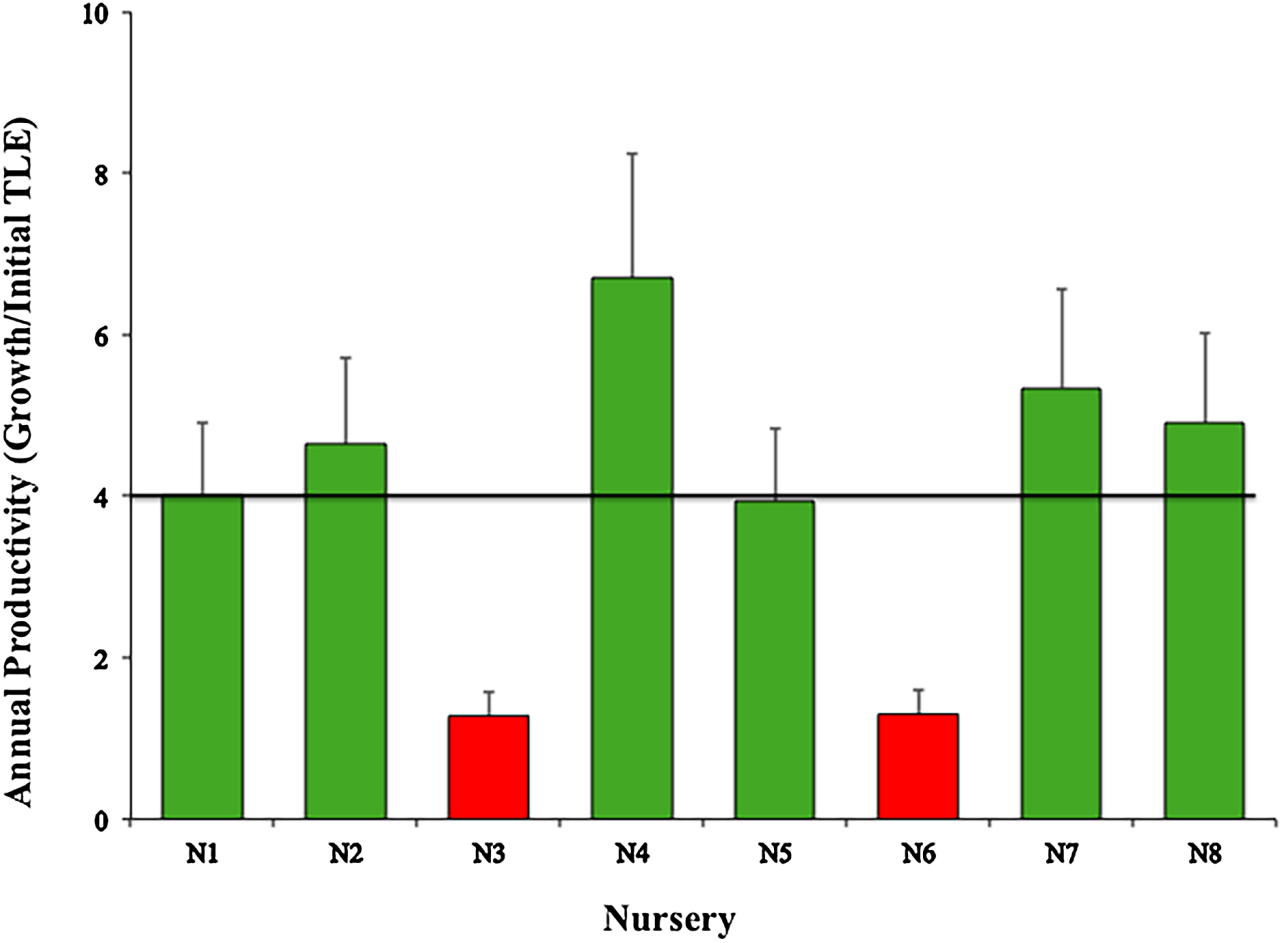
Annual productivity mean values (± SD) of *Acropora cervicornis* in eight coral nurseries. Green bars indicate that they are within 10% of the overall mean, red bars indicate that they are <20% below the mean. Overall mean is indicated by the black line.

The mean productivity value was 4.01 ± 1.88 cm year^−1^ for the eight nurseries (Fig. 5). This study did not evaluate the differences in growth metrics for fragment size (large, medium, or small), or the type of platform (floating or fixed).

### Survival and growth dynamics of outplanted corals

**Figure 6:**
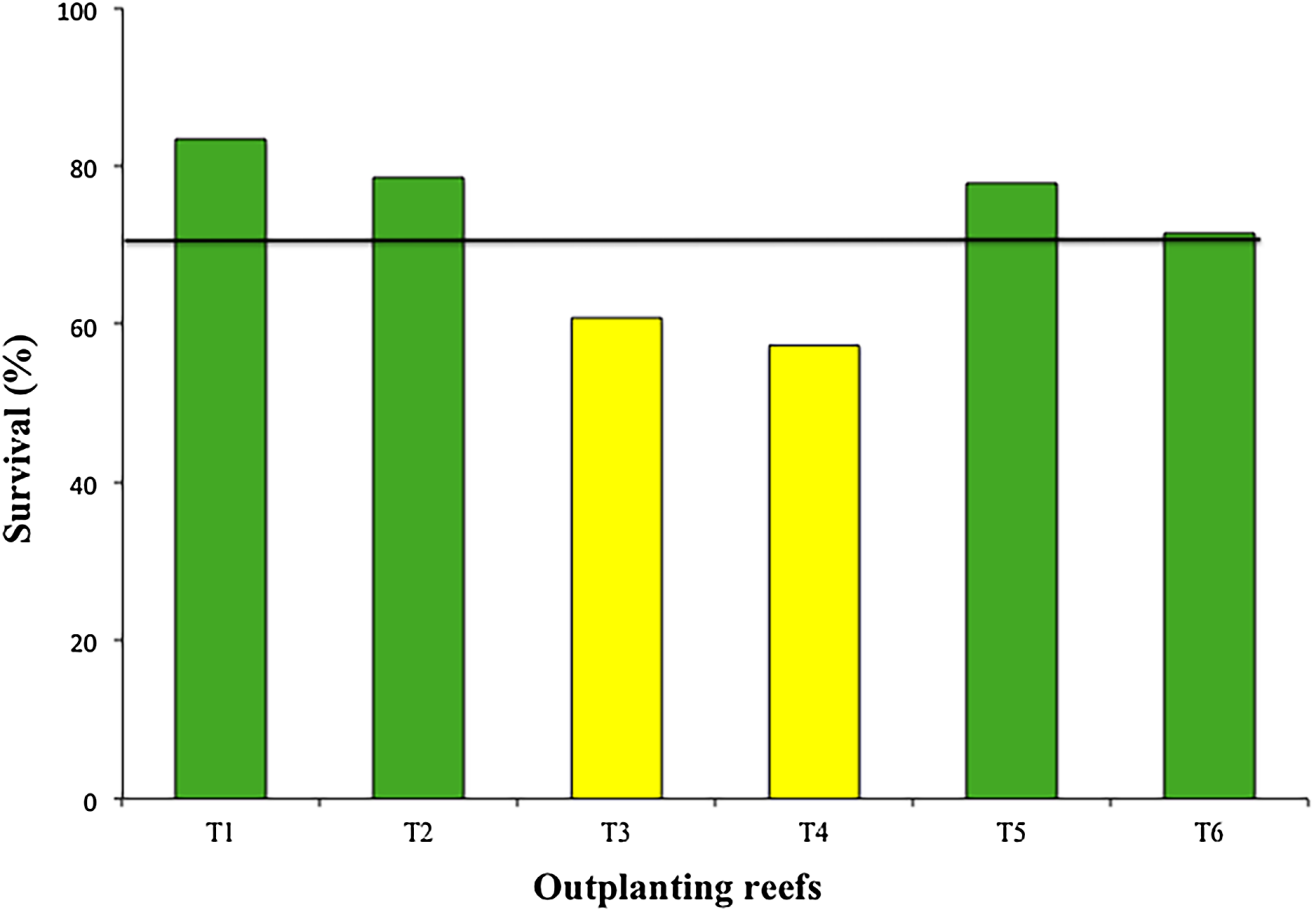
Percentage of survival of colonies in six outplanting sites after 1 year of follow-up. Green bars indicate that they are within 10% of the overall mean, yellow bars that are between 10% and 20% below the mean. Overall mean indicated by the black line.

The mean survival of the six outplanted sites during the 12-month period was 71.55 ± 10.4%, with a range of 57.3–83.3% (Fig. 6). The most common cause of mortality during this period was sedimentation and predation by the fireworm, *Hermodice carunculata*.

**Figure 7:**
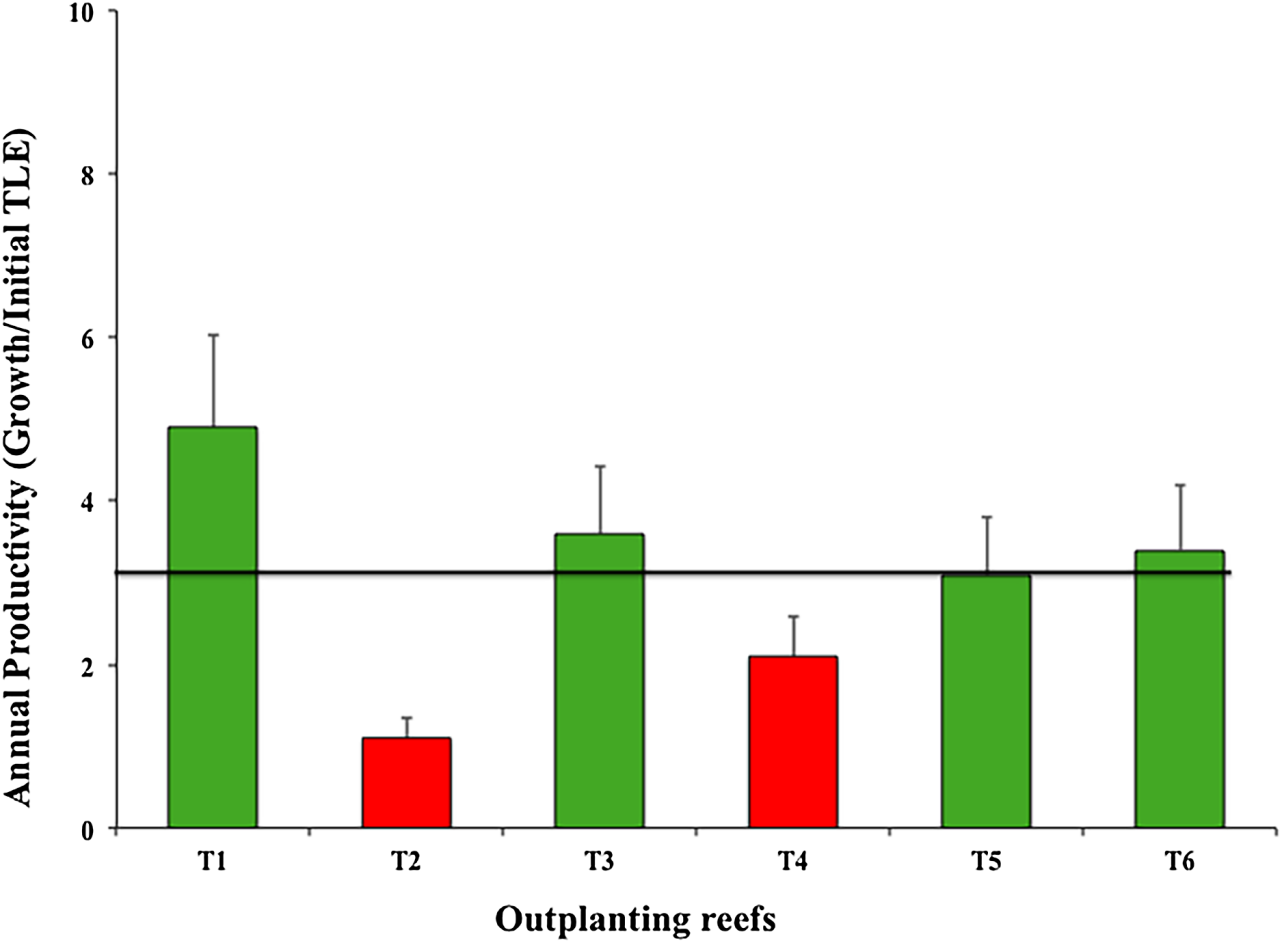
Annual mean productivity values (±SD) of *Acropora cervicornis* in six outplanting sites. Green bars indicate that they are within 10% of the overall mean, red bars indicate that they are <20% below the mean. Overall mean is indicated by the black line.

The six outplanted sites’ mean productivity value was 3.03 ± 1.30 cm year^−1^; of these, T2 and T4 were the least productive (Fig. 7).

### Survival in nurseries and outplanted sites after the strong cyclonic seasons of 2016 and 2017

Cyclonic activity was substantially high in 2016 and 2017, and three hurricanes impacted the study zone: category 4 Matthew (2016), and Irma & Maria (2017), both category 5. Hurricane Matthew caused damage to many of the nursery structures (N1 = −10, N2 = −7, N3 = −7, N4 = −1, N5 = 0, N6 = −5, N7 = −1, N8 = 0), with a loss of 35 structures in total in 2016.

**Figure 8:**
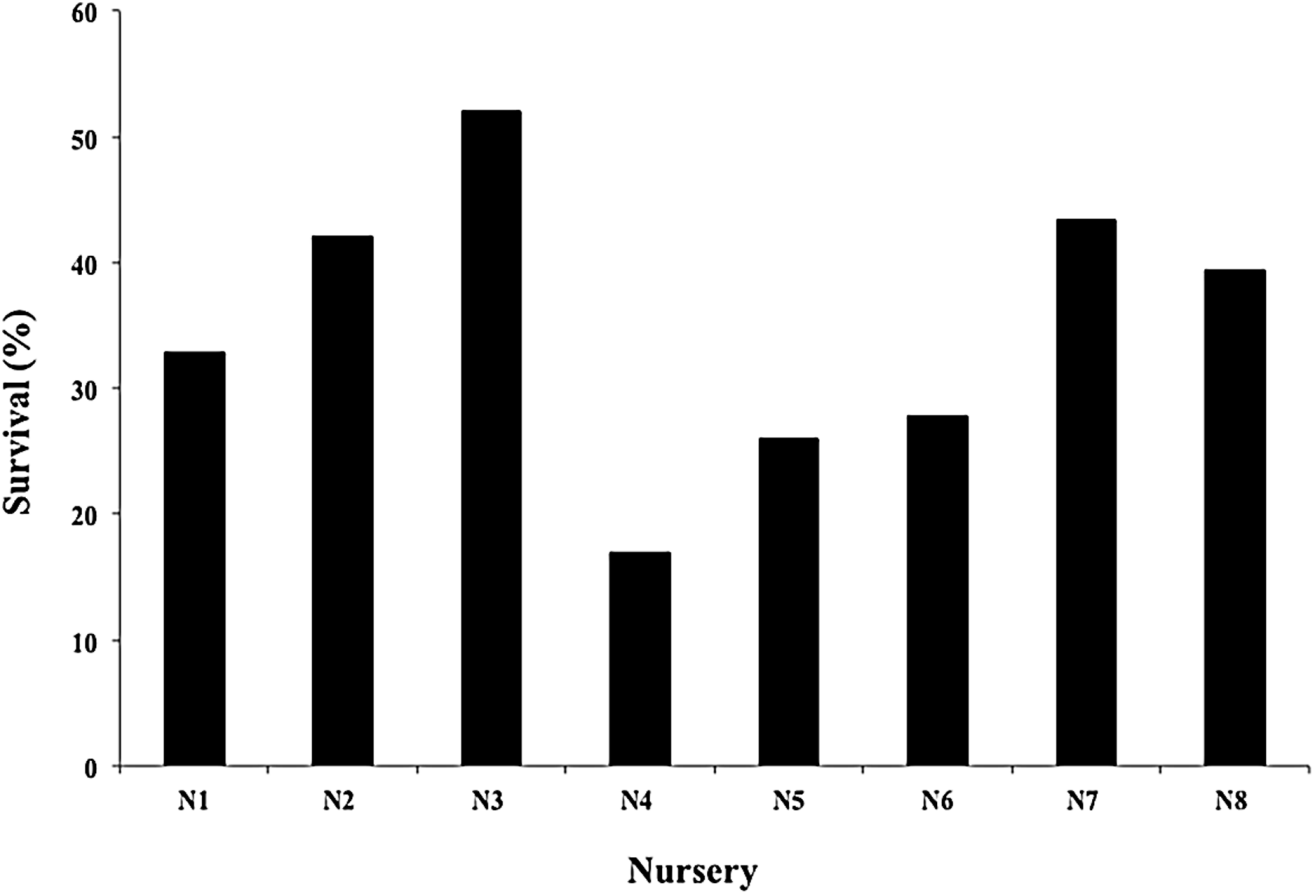
Survival percentage of *Acropora cervicornis* fragments in eight coral nurseries after Hurricanes Matthew (2016), Irma & Maria (2017).

**Figure 9:**
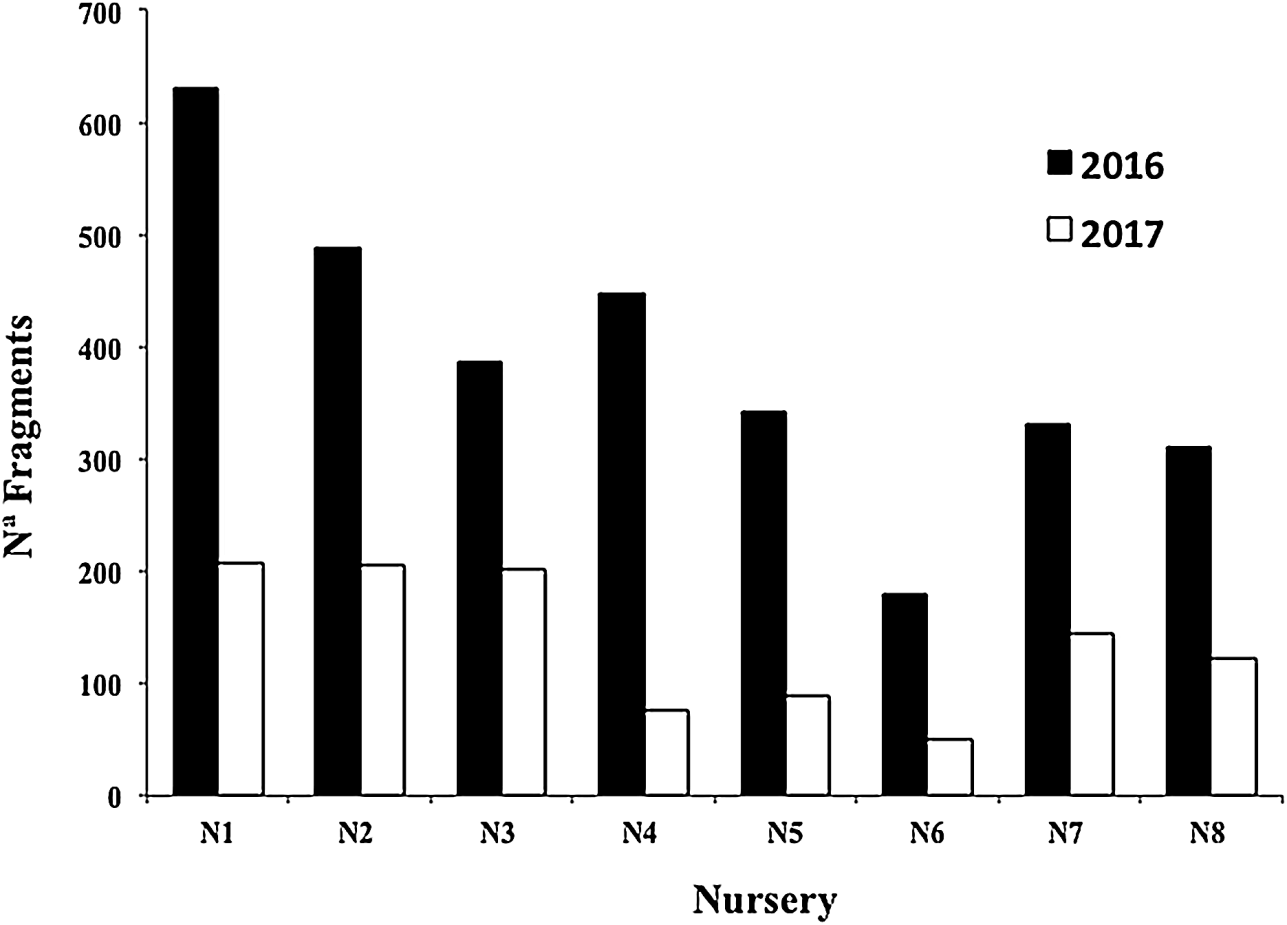
Number of *Acropora cervicornis* fragments in eight coral nurseries after Hurricanes Matthew (2016), Irma & Maria (2017).

The mean survival of all nursery fragments after the 2016 and 2017 cyclonic seasons was 35.06 ± 11.30%, with a range of 16.96–52.07% (Fig. 8). The main cause of mortality was the loss of nurseries structures, which hampered fragment rescue (Fig. 9).

**Figure 10:**
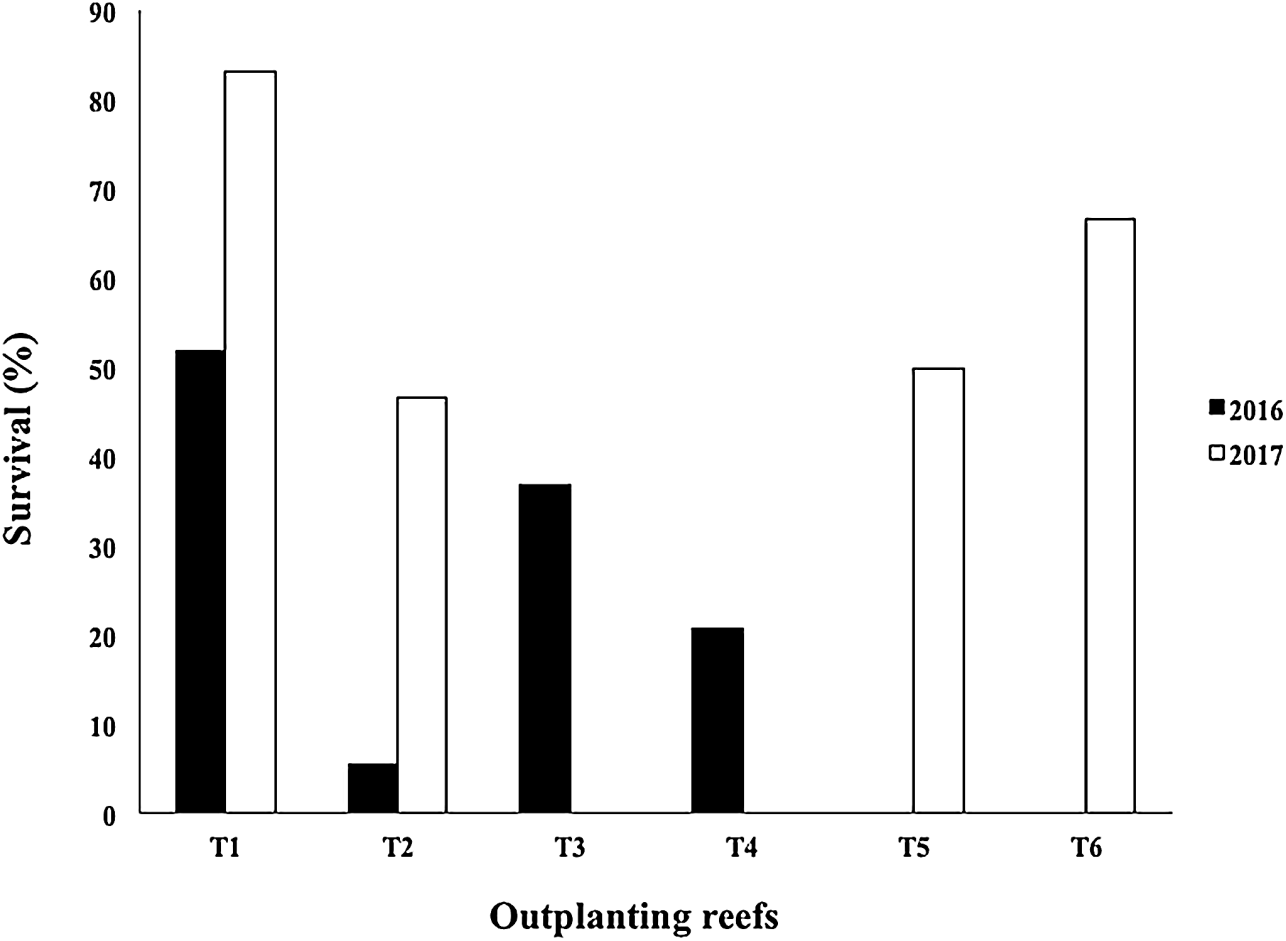
Survival percentage of *Acropora cervicornis* colonies in four outplanting sites (T1, T2, T3, T4) existing after Hurricane Matthew (2016), and four outplanting sites (T1, T2, T5, T6) existing after Hurricanes Irma & Maria (2017).

The mean survival of the outplanted colonies in four outplanted sites operating after Hurricane Matthew (2016) was 28.68 ± 20.0%, with a range of 5.49–51.78%. Due to damage sustained from Mathew in 2017, the T3 and T4 outplanting sites were closed by the program managers. However, T2 was rehabilitated with fragments rescued from the same area, and two new outplanted zones were created (T5 and T6). The mean survival of the outplanted colonies after Hurricanes Irma & Maria (2017) was 61.57 ± 16.86%, with a range of 46.66–83.17% (Fig. 10).

### Coral genotype identity and genetic diversity

The results of our genetic analyses showed that the main nursery contained 32 MLGs of 145 sampled colonies, had a richness of Ng/N = (32/145) = 0.22, and a clonal richness index of (Ng−1)/(N−1) = 0.007.

## Discussion

To assess the performance of the *A. cervicornis* restoration program in the Dominican Republic, we evaluated the growth and survival of nursery and outplanted corals between 2011 and 2017. Our analysis and interpretations were based on the relative yielding (mean) of each nursery and outplanted zone, based upon the stoplight model proposed by Schopmeyer et al. (2017). We documented the results of the program during non-stress conditions and under stress caused by the strong 2016 and 2017 cyclonic seasons in Bayahibe. Additionally, the genotype characterization of coral propagated in nurseries suggest the presence of enough genetic diversity to continue program development.

### Survival and productivity in coral nurseries

Our results showed high survival (>80%) for 12 months across the eight nurseries. This indicates that the standards used for selecting coral nursery farming sites were appropriate. The methods used to propagate corals (transport, structuring, implementation) have efficiently promoted survival and productivity and did not cause mortality. The frequency and methods used for maintenance and monitoring during the first year were also appropriate.

The annual productivity values for the six nurseries were >4.4 cm per year (suggested by Schopmeyer et al. (2017) for Florida and Puerto Rico). Our results confirmed that the yield of each of these nurseries was optimal and growth rates were higher than those reported for wild Staghorn coral (Shinn, 1966; Lewis et al., 1968; Gladfelter & Gladfelter, 1978; Gladfelter, 1984; Tunnicliffe, 1983; Lirman et al., 2010), fulfilling the main objectives of the nurseries to maximize growth rates and minimize mortality (Edwards, 2010).

However, considering we randomly collected the genotypes used for all nurseries and used the same propagating structures (fixed and floating), nurseries N3-Dreams (1.28) and N6-Catalina (1.30) had the poorest performances in terms of coral growth (Fig. 5) (i.e., >4.4 cm per year), indicating that the sites chosen for these two nurseries did not foster coral growth. These results may be due to the fact that the N3 nursery site had low water circulation and high sedimentation, factors that may influence coral growth (Edwards, 2010; Lirman et al., 2014). After Hurricane Matthew, N3 was moved a few meters offshore so its corals could increase their growth rates. The N6-Catalina nursery site receives a large number of daily tourists and watershed discharges with large quantities of sediments, nutrients, and urban wastes from La Romana city (Cortés-Useche et al., 2019).

N6 was one of the nurseries most affected by Hurricane Matthew, losing 84% of its structures. Since it is a shallow and unprotected site, it had a survival rate of 15% after the 2017 hurricane season. Additionally, the time between these two hurricane seasons was very short, and the surviving corals failed to adapt and recover.

Three of the eight nurseries and two of the four outplanted sites suffered significant damage from the strong cyclonic seasons. Although the survival of the eight nurseries averaged 35.07%, our results are encouraging when compared to the mortality reported for Puerto Rico’s nurseries and outplanted sites (>90%) after Hurricanes Irma and Maria (Toledo-Hernández et al., 2018; conv. per. Sean Griffin Restoration Program Puerto Rico NOAA-National Oceanic and Atmospheric Administration, 2017).

Our results indicate that our coral nurseries are genotype reservoirs better adapted to the strong environmental changes occurring in 2016 and 2017. Nurseries have served as havens in the face of disease outbreaks, storms, and extreme temperatures (Edwards, 2010; Johnson et al., 2011; Griffin et al., 2012; Schopmeyer et al., 2012; Rinkevich, 2015). They also serve as production sites for coral larvae, fishes, and other organisms (Amar & Rinkevich, 2007; Shafir & Rinkevic, 2010), contributing to overall ecosystem diversity.

In 2015 and 2016, restoration activities were supplemented by assisted fertilization, suggesting that *A. cervicornis* colonies from the main nursery can reach sexual maturity and release their gametes (Calle-Triviño et al., 2018). Likewise, gametes and larvae raised from nursery populations can provide key resources for research on assisted evolution and genetic engineering (Rinkevich, 2014; Van Oppen et al., 2015; Lirman & Schopmeyer, 2016). Nurseries can generate thousands of planula larvae to act as larvae dispersion centers (Rinkevich, 2015), which in turn could establish larvae connectivity routes between coral patches (Calle-Triviño et al., 2018).

Our data indicate that genetically diverse populations within a nursery are valuable due to the assisted fertilization success they provide to nursery and outplanted stock. It should be noted that additional studies and information are needed to determine the compatibility of the known genotypes and the success of their offspring.

### Survival and productivity in outplanted sites

The main objectives of the nursery phase include minimizing coral mortality and maximizing productivity. However, outplanted sites’ primary purpose is to establish genetically diverse populations (Lirman & Schopmeyer, 2016).

The challenge is to ensure that degraded reefs increase their structural complexity by outplanting corals that have been raised in nurseries. These corals can reproduce sexually, thereby increasing genetic diversity and support for the establishment of other species. We expect the formation of biological corridors, essential for ecosystem connectivity and indispensable for increasing functional biodiversity and reef resilience (Drury, Manzello & Lirman, 2017).

Our results showed high survival rates (>70%) during the first 12 months for four of the six outplanted sites. These rates match the benchmark proposed by Schopmeyer et al. (2017) for the survival of outplanted corals during the first year). Only two of the outplanted sites (T3 and T4) were >10% lower than the mean. Mortality in these two zones was associated with the presence of predators, mainly fireworms (Calle-Triviño et al., 2017). These two zones are adjacent, separated by less than 500 m. It is possible that ineffective maintenance and cleaning of these two zones allowed the fast growth and spread of fireworms.

As for annual productivity, four outplanted zones were within the benchmark (4.8 cm year^−1^) suggested by Schopmeyer et al. (2017) for Florida and Puerto Rico. However, two outplanted sites were at high risk (i.e., <4.8 cm per year), indicating that the selected sites did not provide a favorable environment for coral establishment and growth. Zone T2-Coralina is particularly vulnerable because it is very close to the urban zone (500 m) and is thus directly impacted. Moreover, boats travel through and dock in the area. Although water in this zone is in constantly moving and circulating, the site is shallow (about 2–5 m). The fragment genotypes were collected in deeper areas and were maintained at the same depth in nurseries. We could not predict the nursery yield because it did not always correlate with yield of the outplanted sites. Additionally, genotypes may have very different growth rates in different environments (Lirman et al., 2014; Drury, Manzello & Lirman, 2017). T2-Coralina was the zone most affected by the strong 2016 and 2017 cyclonic seasons.

### Coral genotype identity and genetic diversity

Our results suggest that the main nursery had higher genotypic diversity (32 different genotypes) compared to other nurseries in the Dominican Republic (13 genotypes in FEPC) and Florida (24 genotypes). The high genotypic diversity represented within this nursery, as well as the compatibility of those genotypes successfully demonstrated by the assisted fertilization initiatives in 2015 and 2016 (Calle-Triviño, Arias-González & Sellares, 2016; Calle-Triviño et al., 2018), confirm that the restoration program in the Dominican Republic should be expanded to maintain and increase diversity.

These results are ecologically important because the main nursery populations represent a functional unity (source of coral) for coral reef recovery through an active conservation response (Drury, Manzello & Lirman, 2017). Coral can be very useful in increasing genetic diversity and population density when outplanted to degraded or disturbed sites, and they can also contribute to increased sexual reproductive success (Quinn & Kojis, 2006; Vollmer & Palumbi, 2007; Baums, 2008; Bowden-Kerby, 2008; Griffin et al., 2012).

The dominant reproduction mode of a specific population of species is crucial as it influences environmental stress management with long-term permanence. This must be considered when developing management and restoration strategies to protect and preserve species (Baums, 2008). The distribution of the clonal individuals identified in the main nursery, as well as the high diversity of genets found in this study, suggest that genotypes can help develop and improve the restoration program in the southeastern part of the island (Baums et al., 2013; Iwao et al., 2014; Miller et al., 2017; Baums et al., 2019). Different genotypes planted with enough proximity can allow cross-fertilization during massive spawning events. This is also relevant for restoration programs.

Moreover, restoration efforts should include information on the management and handling of species produced by genetic studies. Genetic patterns can guide conservation actions to obtain more resistant individuals able to cope with dramatic environmental changes, diseases, and pollutants, thus increasing genetic viability and preserving adaptive potential (Drury, Manzello & Lirman, 2017). Our genotype identification established a baseline that will allow for the future spatially distributed selection of colonies in outplanted sites (Baums, 2008; Schopmeyer et al., 2012). This is also useful for future studies on genotype resistance against different stressors, such as high sedimentation, temperature increase, and predation by fireworms (Lundgren et al., 2013; Zayasu, Satoh & Shinzato, 2018).

Our work documented the growth and survival of *A. cervicornis* coral nurseries and outplants in southeast Dominican Republic. We believe that working together with researchers, practitioners, students, community volunteers, environmental authorities, and the tourism industry creates a higher level of coral reef conservation efforts in the region. We recommend that these alliances be strengthened for the sake of coral reefs, and that the systematic long-term monitoring of outplanted sites be continued, to build a scientific model helpful for studying spawning, improve the understanding of current functional aspects of these habitats, and provide information on the system’s stability and resilience.

## Conclusions

When considering the predicted persistence, recovery, and extinction risk of *A. cervicornis*, intrinsic characteristics and external threats are important factors to consider. This species is at risk because of its continuous decline in abundance and the permanence of its threats (Harvell et al., 1999; Hoegh-Guldberg, 1999; Weil, 2004; Gardner et al., 2005).

Although *A. cervicornis* has persisted at extremely low levels of abundance, the recovery of this species may not be possible due to the permanence of its stressors. Therefore, active restoration efforts like the one described in this study are necessary.

The restoration program examined in this case study has provided a number of benefits for the local ecosystem and economy, such as: (1) maintaining genetic diversity in nurseries with 32 available genotypes, (2) creating outplanted sites that have contributed to the rapid creation of fish and invertebrate habitats that would otherwise take decades to form, (3) providing a sustainable source of corals for experimental research, and (4) providing unique volunteer and employment opportunities for local communities interested in participating in the restoration process.

We believe that the regional restoration benchmarks for *A. cervicornis* proposed by Schopmeyer et al. (2017) can be widely applied in the comparison of programs across the Caribbean.

## Supplemental Information

10.7717/peerj.8863/supp-1 Supplemental Information 1 Allelic Frequencies (*N* (clones removed) = 32) for *Acropora cervicornis* in the “mother nursery”. Na = number of different alleles, Ne = number of effective alleles, I = Shannon index, Ho = Heterocigosis observed, He = Heterocigosis expected, UHe = unbiased expected heterozygosity, F = fixation index (GenAlEx v 6.41).

10.7717/peerj.8863/supp-2 Supplemental Information 2 Raw data exported from Excel and genalex for data analyses for Heterozygosity, Fstatistics and Polymorphism by Locus for Codominant Data and preparation for Table S1.

10.7717/peerj.8863/supp-3 Supplemental Information 3 Raw data exported from Excel and genalex for data analyses of and preparation for Probability of Identity (PI) for each Locus and for Increasing Combinations of the 4 Loci and preparation for Table S1.

10.7717/peerj.8863/supp-4 Supplemental Information 4 Raw data exported from GENECLONE for Distinct MLG designation.

10.7717/peerj.8863/supp-5 Supplemental Information 5 Raw data exported from GENECLONE for MLGs distant and somatic mutation.

10.7717/peerj.8863/supp-6 Supplemental Information 6 Raw data exported from Excel and Genalex for early data exploration and analysis.

10.7717/peerj.8863/supp-7 Supplemental Information 7 Raw data exported from Excel for data analyses and preparation for Figures 4-10.

## Additional Information and Declarations

### Field Study Permissions

The following information was supplied relating to field study approvals (i.e., approving body and any reference numbers): The Ministry of Environment and Natural Resources, Vice Ministry of Protected Areas and Biodiversity, Directorate of Biodiversity and Wildlife approved field collection (DO-0040). An aquaculture import certificate was granted by the Secretary of Agriculture, Livestock and Rural Development, Fish and Food, National Service of Health, Safety and Food Quality, General Directorate of Phytozoosanitary Inspection (CIT072016033578).

### Data Availability

The following information was supplied regarding data availability: The raw and processed gen clone data are available in the Supplemental Files.
