# Supplementary material for: Assessing and genotyping threatened staghorn coral Acropora cervicornis nurseries during restoration in southeast Dominican Republic
Source: PeerJ. 2020 Apr 17;8:e8863. doi: 10.7717/peerj.8863 (PMC7169967; doi:10.7717/peerj.8863)
Supplement: Supplemental Information 1 — Na = number of different alleles, Ne = number of effective alleles, I = Shannon index, Ho = Heterocigosis observed, He = Heterocigosis expected, UHe = unbiased expected heterozygosity, F = fixation index (GenAlEx v 6.41). [file peerj-08-8863-s001.docx]

**Table 3_Suplementary:**

**Allelic Frequencies (N (clones removed) = 32) for *Acropora cervicornis* in the "mother nursery".**

Na = number of different alleles, Ne = number of effective alleles, I = Shannon index, Ho = Heterocigosis observed, He = Heterocigosis expected, UHe = unbiased expected heterozygosity, F = fixation index (GenAlEx v 6.41).

| **Locus** | ***Na*** | ***Ne*** | ***I*** | ***Ho.*** | ***He*** | ***uHe*** | ***F*** |
| --- | --- | --- | --- | --- | --- | --- | --- |
| 166 | 11.00 | 3.85 | 1.8 | 0.62 | 0.74 | 0.75 | 0.15 |
| 181 | 11.00 | 2.3 | 1.36 | 0.43 | 0.56 | 0.57 | 0.22 |
| 182 | 13.00 | 8.19 | 2.29 | 0.75 | 0.87 | 0.89 | 0.14 |
| 207 | 13.00 | 7.84 | 2.25 | 0.62 | 0.87 | 0.88 | 0.28 |
| Mean (SE) | 12.00 (0.57) |  |  |  |  |  |  |
